# Carbon allocation of *Spirodela polyrhiza* under boron toxicity

**DOI:** 10.3389/fpls.2023.1208888

**Published:** 2023-07-17

**Authors:** Débora Pagliuso, João Pedro de Jesus Pereira, João Cristiano Ulrich, Marycel Elena Barbosa Cotrim, Marcos S. Buckeridge, Adriana Grandis

**Affiliations:** ^1^ Laboratory of Plant Physiological Ecology, Department of Botany. Institute of Biosciences, University of São Paulo, São Paulo, Brazil; ^2^ Nuclear and Energy Research Institute, University of São Paulo, São Paulo, Brazil

**Keywords:** duckweed, sugar, polysaccharides, pectin, apiose

## Abstract

Pectic polysaccharides containing apiose, xylose, and uronic acids are excellent candidates for boron fixation. Duckweeds are the fastest-growing angiosperms that can absorb diverse metals and contaminants from water and have high pectin content in their cell walls. Therefore, these plants can be considered excellent boron (B) accumulators. This work aimed to investigate the relationship between B assimilation capacity with apiose content in the cell wall of *Spirodela polyrhiza* subjected to different boric acid concentrations. Plants were grown for 7 and 10 days in ½ Schenck-Hildebrandt media supplemented with 0 to 56 mg B.L^-1^, the non-structural and structural carbohydrates, and related genes were evaluated. The results showed that B altered the morphology and carbohydrate composition of this species during plant development. The optimum B concentration (1.8 mg B.L^-1^) led to the highest relative growth and biomass accumulation, reduced starch, and high pectin and apiose contents, together with increased expression of UDP-apiose/UDP-xylose synthase (*AXS*) and 1,4-α-galacturonosyltransferase (*GAUT*). The toxic state (28 and 56 mg B.L^-1^) increased the hexose contents in the cell wall with a concomitant reduction of pectins, apiose, and growth. The pectin content of *S. polyrhiza* was strongly associated with its growth capacity and regulation of B content within the cells, which have *AXS* as an important regulator. These findings suggest that duckweeds are suitable for B remediation, and their biomass can be used for bioenergy production.

## 1 Introduction

Duckweeds are the smallest flowering aquatic plants on Earth (Landolt, 1992), encompassing 36 species divided into five genera: *Spirodela, Landoltia, Lemna, Wolffiella*, and *Wolffia* (Les et al., 2002; Bog et al., 2019). Their fast-growing capacity and high molecular performance to absorb essential metals and metalloids from the water make duckweeds interesting for comprehending the allocation and assimilation of those compounds into the plant biomass (Oron et al., 1987; Davis et al., 2002; Ziegler et al., 2014). In addition, duckweed growth is 30 times faster than any other crop species (Lam et al., 2014), which might be related to the plant’s ability to capture the essential micronutrient boron (B), which still lacks validation.

Among plants that accumulate B, duckweed is considered an excellent boron accumulator (780 mg B per kg of cell wall) (Glandon and Mcnabb, 1978; Frick, 1985; Matoh, 1997). B is strictly associated with its ability to form dimers covalently cross-linked by borate esters with the hydroxyl groups of cell wall carbohydrates and/or glycoproteins (O’Neill et al., 1996; Bharadwaj et al., 2020). Boron association with apiose has a computational model to describe its cross-linking; however, it is not known with the stereoisomer generated in plants (Bharadwaj et al., 2020). Nevertheless, B can also bind to smaller proportions of non-specific polysaccharides (Matsunaga et al., 2004). Plant cell walls are a complex structure of polysaccharides, proteins, and phenolic compounds organized in a cellulose core cross-linked to hemicelluloses and lignin, immersed in a pectin matrix (Carpita and Gibeaut, 1993).

Duckweeds have a distinct cell wall composition with high levels of pectin (50% of the cell wall), a complex polysaccharide class built up from galacturonic acid chains with substitutions and ramifications of different sugars (Mohnen, 2008). These plants have elevated content of apiogalacturonan and xylogalacturonan, pectins rich in apiose and xylose, respectively (Avci et al., 2018; Sowinski et al., 2019; Pagliuso et al., 2020; Pagliuso et al., 2022). Pectic polysaccharides containing apiose, xylose, and uronic acids are excellent candidates for B fixation (Matoh, 1997). Apiose is a five-carbon branched sugar with a furanoid ring preferred to stabilize the borate ester complex formed (Watson and Orenstein, 1975; Brown et al., 2002). Therefore, pectins may be related to the capacity to retain low B concentrations available in the water in the plant tissues for their growth and development.

B occurs naturally as borosilicates, boric acid, borax, and other borate minerals that are mobilized to plants by weathering (Davis et al., 2002). In water, B occurs as free borate, polyborates, and complex with transition metals (Bassett, 1980). B availability in water is low (0.1–1 mg B L^−1^) (Wyness et al., 2003) and related to natural factors (weathering of rocks, leaching salt deposits, sea salt, and rainfall), industrial applications (such as glass and ceramic manufacture, insulation products, agrochemicals, and detergents), and effluents disposal of the drainage from coal mines, mining industry, and oil refinery due to the lack of legal regulation (Adriano et al., 1980; Jahiruddin et al., 1998). The several applications of B in the industry are a concern, particularly regarding its untreated wastewater due to the environmental phytotoxicity, teratogenic reproduction, and growth effects (Landauer, 1952; Butterwick et al., 1989; Smith and Anders, 1989; Davis et al., 2003; Camacho-cristóbal et al., 2008). Besides that, B availability may affect several metabolic pathways in plants, which leads to changes in physiological and morphological features involving cell wall synthesis and structure maintenance (cell size, rigidity, expandability, porosity, and tension strength) (Matoh, 1997; Blevins and Lukaszewski, 1998; Camacho-cristóbal et al., 2008; Riaz et al., 2021). This metal toxicity may reduce cell division, as well as lower lignin, suberin, chlorophyll contents, and photosynthetic activity (Camacho-cristóbal et al., 2008). Other changes are found in the cell wall by boron toxicity, mainly regarding the pectins. The content of uronic acids and total pectin is reduced along with changes in crosslinks and alteration of pectin methylesterase activity (Wu et al., 2018; Yan et al., 2021). There are also alterations in hemicellulose and cellulose architecture (Liu et al., 2014; Wu et al., 2018; Riaz et al., 2021). Evidence by FTIR suggests that boron toxicity cleavage hydrogen bonds between protein, cellulose, and hemicellulose and that the cellulose levels are reduced in rice seedlings and navel oranges (Liu et al., 2014; Riaz et al., 2021). The evaluation of boron toxicity in a transcriptome of *Arabidopsis thaliana* shoots shows differently expressed transcripts related to pectin synthesis and NDP-sugars together with an increase of arabinose, galactose, rhamnose, xylose, mannose, fructose, and glucose, suggesting the capture of excess boron by the cell wall carbohydrates (Wang et al., 2021). Furthermore, the B requirement for 14 species was related to the pectin content and composition, correlating positively with the sugar’s uronic acid, rhamnose, and galactose contents (Hu et al., 1996). These strong correlations and the duckweed cell wall rich in pectins make those plants interesting models to evaluate the cell wall alterations and the importance of apiose in boron toxicity. Studies evaluating B tolerance and toxicity in *Spirodela polyrhiza* and *Lemna minor* revealed that plants show necrotic and chlorotic fronds, reduction in relative growth, and plant death in B concentrations ranging from 0 to 40 mg L^−1^ (Davis et al., 2002; Villavicencio et al., 2007; Türker et al., 2016), but no information about the cell wall composition and characterization is found. The present work demonstrated the importance of B to *S. polyrhiza* development, involved in controlling carbon allocation and cell wall composition, especially pectin-related compounds.

## 2 Materials and methods

### 2.1 Plant growth material

*Spirodela polyrhiza* 9509 (ecotype from Stadtroda, Lotchen, Germany) was obtained as *in vitro* culture from the Rutgers Duckweed Stock Cooperative (RDSC) collection and was cultivated under axenic conditions in 250-ml borosilicate erlenmeyers with 100 ml of in ½ Schenk-Hildebrandt (SH) medium (Sigma-Aldrich^®^) containing 2 M (NH₄)₃PO₄, 0.08M H_3_BO_3_, 1.28 M CaCl_2_, 0.42 mM CoCl_2_•6H_2_O, 1.25 mM Cu.SO_4_*5H_2_O, 0.05 M Na_2_-EDTA, 0.05 FeSO_4_•7H_2_O, 1.62 M MgSO_4_, 0.06 mM MnSO_4_*H_2_O, 1.28 mM H_2_MoO_3_•2H_2_O, 6 mM KI, 22.7 M KNO_3_, and 3.5 mM ZnSO_4_•7H_2_O (pH 6.5), and supplemented with 0.5% sucrose. Then, the standard ½ SH was modified by adding different concentrations (0, 0.4, 0.9, 1.8, 3.5, 7, 14, 28, and 56 mg B L^-1^) of boric acid (17.49% B–MW 61.83 g mol^−1^, 99.5%) to evaluate the effect of this micronutrient on duckweed growth. The boron concentrations correspond to 0.007, 0,014, 0.028, 0.056, 0.112, 0.450, and 0.900 M of boron, and the DI water used in media preparation has no boron content. The regular growth is performed with 0.4 mg L^−1^ B, used as a control Appenroth (2015). Twenty-five fronds of *S. polyrhiza* were grown for 7 and 10 days at 25°C with a photoperiod of 16 h of light (photosynthetic active radiation intensity of 100 µmol m^−2^ s^−1^). At each harvest (7 and 10 days), the fresh biomass was weighed, frozen in liquid nitrogen, and ground to a fine powder with a mortar and pestle. Samples were stored at −80°C and freeze-dried for molecular and biochemical analyses.

### 2.2 B quantification and pH evaluation

B remaining in the culture media and B content in the duckweed biomass were quantified after the disposal in the borosilicate erlenmeyers; therefore, if there was any leak, it was incorporated in the mensuration. At each harvest (after 7 and 10 days of growth), the media pH was measured with a pH meter (Metler Toledo Seven Compact), and 5 ml of culture media was frozen at −20°C to evaluate B concentration by inductively coupled plasma-optical emission spectrometry (ICP-OES, Spectro ARCOS, AMETEK). To determine B content in biomass, 50 mg of fresh biomass was digested using nitric acid (Liu et al., 2018). To each sample, 5 ml of 65% nitric acid and 1 ml of 30% hydrogen peroxide were added and heated at 90°C for 4 h. The final reaction was diluted to a concentration of 8% nitric acid, filtered at 0.22 µM, and stored at −20°C. B concentration was determined by ICP-OES and quantified by comparison with a calibration curve (0.1 to 5.0 µg g^−1^ of B single element, Inorganic Ventures).

### 2.3 Growth rate

The plants’ relative growth rate (RGR) was calculated according to the International Steering Committee on Duckweed Research and Applications (ISCDRA). Growth measurements followed the procedure described by Ziegler et al. (2014). Twenty-five fronds of *S. polyrhiza* were initially inoculated into the culture medium, corresponding to one biological replicate. Simultaneously, five replicates were harvested (*t*_0_, first day of the experiment). RGR was calculated by Equation 1, simplified into Equations 2 and 3, where *x* represents the data of fresh biomass and t represents elapsed time in days (zero = *t*_0_, 7 days = *t*_7_, and 10 days = *t*_10_).


(1) $$X_{t}=\;x_{t0}*e^{RGR*t\;}$$

(2) $$RGR=\;\frac{lnx_{t7}-lnx_{t0}}{t_{7}-t_{0}}$$

(3) $$RGR=\;\frac{lnx_{t10}-lnx_{t0}}{t_{10}-t_{7}}$$

### 2.4 Non-structural carbohydrates

Soluble sugars were extracted three times from 10 mg of powdered dry biomass with 1.5 ml of 80% ethanol (v/v) at 80°C for 20 min each. The supernatants containing the soluble sugars were recovered by centrifugation at 14,000 rpm for 10 min, vacuum concentrated (ThermoScientific^®^ Savant SC 250 EXP), and resuspended in 1 ml of deionized water with 1 ml of chloroform to remove pigments. Sucrose, fructose, glucose, and raffinose were analyzed by high-performance anion exchange chromatography with pulsed amperometric detection (HPAEC-PAD) in a Dionex^®^ system (ICS 5,000) using a CarboPac PA1 column and eluted with 150 µM sodium hydroxide in an isocratic run of 27 min (Pagliuso et al., 2018).

The alcohol-insoluble residue (AIR) was dried overnight at 45°C before starch digestion (Arenque et al., 2014). Starch was solubilized with 120 U ml^−1^ of α- amylase (E.C. 3.2.1.1) of *Bacillus licheniform*is (Megazyme^®^) in 10 mM MOPS buffer (pH 6.5) at 75°C for 1 h. Afterward, 30 U ml^−1^ of amyloglucosidase (E.C. 3.2.1.3) of *Aspergillus niger* (Megazyme^®^) in 100 mM sodium acetate (pH 4.5) was added, and the mixture was incubated at 50°C for 1 h. To the recovered supernatants, a mixture containing glucose oxidase (1,100 U ml^−1^), peroxidase (700 U ml^−1^), 4-aminoantipirin (290 µmol L^−1^), and 50 mM of phenol at pH 7.5 was added to determine the released glucose by a colorimetric assay. The reactions were further incubated for 15 min at 30°C, and the absorbance was measured at 490 nm.

### 2.5 Structural carbohydrates

Aliquots of 2 mg of de-starched AIR were hydrolyzed with 1 ml of 2 M trifluoroacetic acid for 1 h at 100°C to obtain non-cellulosic monosaccharides. Then, the samples were vacuum dried and resuspended in 1 ml of MilliQ water. Cellulosic hydrolysis was performed with 72% sulfuric acid at 45°C for 30 min, diluted to 4%, and incubated at 100°C for 1.5 h. Samples were filtered through 0.22-µm filters (Millipore^®^). Apiose, arabinose, fucose, galactose, glucose, mannose, rhamnose, and xylose were analyzed by HPAEC-PAD (ICS 5,000 system, Dionex-Thermo^®^) on a CarboPac SA10 column (Dionex-Thermo^®^). Sugars were eluted isocratically with 99.2% of water and 0.8% sodium hydroxide (v/v) (1 ml min^−1^) and detected using a post-column base containing 500 mM NaOH (0.5 ml min^−1^).

### 2.6 Uronic acid determination

Uronic acids were determined as described by Filisetti-Cozzi and Carpita (1991). Aliquots of 5 mg of the de-starched cell wall was hydrolyzed in 2 ml of sulfuric acid and 1 ml of water was added. Each reaction was incubated on ice under stirring (1,250 rpm) for 5 min and diluted to 10 ml. To the 400 µl of the supernatant, 40 µl of 4 M sulfamic acid in potassium sulfamate solution (pH 1.6) and 2.4 ml of 75 mM sodium borate in sulfuric acid were added. The mixture was incubated at 100°C for 20 min. The reactions were cooled on ice for 10 min, and 80 µl of m-hydroxyphenyl in 0.5% NaOH was added for color development. The absorbance was read at 525 nm (Genesys 10S UV-VIS, Thermo Scientific) with a standard curve of 0.12–2.5 M of D-galacturonic acid for inferring the pectin content.

### 2.7 RNA extraction, DNase treatment, and cDNA synthesis

RNA was extracted using the ReliaPrepTM RNA Tissue Miniprep System (Promega^®^) followed by DNase treatment according to the manufacturer’s instructions. The RNA quantification and purity were assured using a Nanodrop ND-100 spectrophotometer (Thermo-Fischer Scientific^®^), and samples with 260/280 ratios between 1.8 and 2.2 were considered sufficiently pure. The samples’ integrity was also checked by electrophoresis on a 1% agarose gel and stained with SYBR Safe DNA gel stain (Thermo-Fischer Scientific^®^). Approximately 1 µg of each RNA sample was reverse transcribed with random hexamers by SuperScript III Reverse Transcriptase (Thermo-Fischer Scientific^®^). The samples were tested for the absence of genomic DNA with UPD-*apiose/UDP-xylose synthase* (*AXS*) primers (Forward: 5’-GCATCCAGTTCCACCGTCTC-3’; Reverse: 5’-GCAGGGCGTTTCATCTTCTTT-3’) as described by Pagliuso et al. (2022).

### 2.8 Targets and qRT-PCR analysis

*S. polyrhiza* 9509 (GenBank assembly accession GCA_001981405.1, loci CP019093.1—CP019112.1) *ab initio* gene prediction was performed with the Augustus prediction tool (version 3.3.2) on an *A. thaliana* gene model (http://augustus.gobics.de/) and the functional annotation was verified by BLASTp, InterProCan, and Gene Ontology as described by Pagliuso et al. (2022). The sequence of sugar and cell wall-related genes from *A. thaliana* were used as queries to *S. polyrhiza* 9509 and the recovered *S. polyrhiza* 9509 genes were compared with the Reference Sequence (RefSeq) database of NCBI by BlastX (*E*-value > e^−10^), InterPro database, and HMMER scan (https://www.ebi.ac.uk/Tools/hmmer/) for protein family association and functional domain validations. Primers were designed with Primer-BlastR (NCBI) (https://www.ncbi.nlm.nih.gov/tools/primer-blast/) according to MIQE guidelines. The primer sequences are shown in **Table S1**.

The relative abundance of target transcripts in *S. polyrhiza* 9509 was measured by qRT-PCR analysis using a QuantiStudio 6 Flex Real-Time PCR system (Applied-Biosystems, Thermo-Fischer Scientific^®^). The PCR reactions were performed with 1.4 µl of cDNA (1:10), 7 µl of 2X SYBR Green Master Mix (Applied-Biosystems, Thermo-Fischer Scientific^®^) 800 nM primer set, and the following cycling conditions: 95°C for 10 min, 40 cycles of 95°C for 15 s, 60°C for 30 s, and 72°C for 30 s. A melting curve analysis confirmed the amplification of a single product. The cycle quantification (Cq) values and the efficiency of each primer were determined using LinRegPCR software (Vandesompele et al., 2002). The relative abundance of *UDP-apiose/UDP-xylose synthase (AXS), UDP-glucuronate decarboxylase (UXS), rhamnose biosynthesis (RHM)*, and *α-galacturonosyltransferase (GAUT)* was quantified compared to the average expression, normalized by the highest Cq value, and the Cq values of the mentioned targets were normalized by the geometric average of the reference gene combinations [*Elongation factor 1-α (EF1)* and *F-box family protein (FBOX)*]. The specific transcripts were selected based on the highest expression levels, as reported by Pagliuso et al. (2022).

### 2.9 Data analysis

Five replicates were used for the relative growth rate, biomass accumulation, boron dosage in media, pH evaluation, soluble sugars, starch, uronic acids, monosaccharide quantification, and gene transcript analyses. Owing to plant biomass availability, three replicates were used for B dosage in plants. Data from each harvest day (7 and 10 days) were evaluated by one-way ANOVA followed by Tukey’s test (*p*< 0.05). In addition, a comparison between 7 and 10 days was made with Student’s *t*-test (*p*< 0.05) for each concentration. The analyses were carried out with R software version 3.6.1.

## 3 Results

### 3.1 B increases growth but is toxic at elevated concentrations

**Figure 1:**
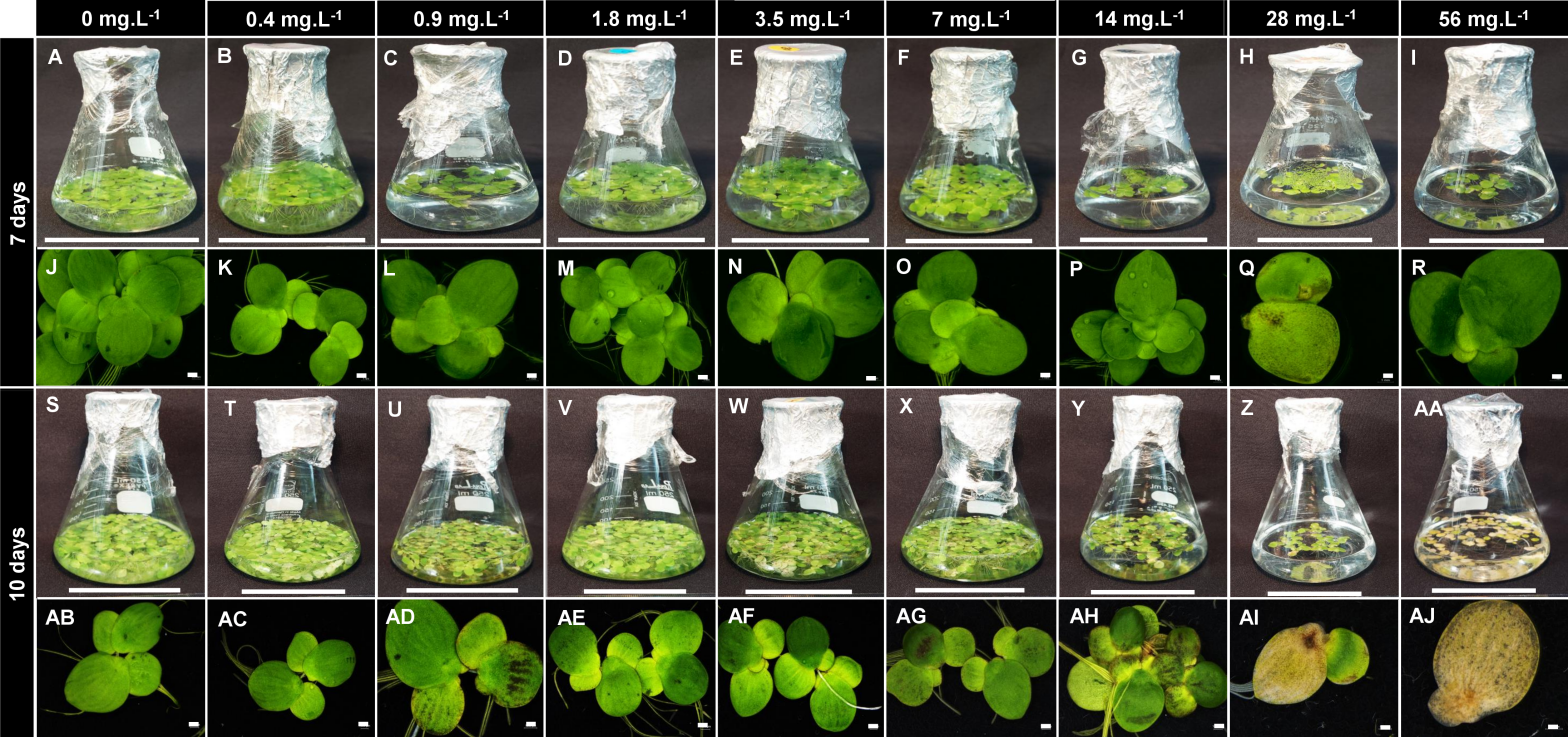
Boron toxicity and deficiency in *Spirodela polyrhiza.* Plants were grown under nine different boron concentrations (0 mg L^−1^; 0.4 mg L^−1^; 0.9 mg L^−1^; 1.8 mg L^−1^; 3.5 mg L^−1^; 7 mg L^−1^; 14 mg L^−1^; 28 mg L^−1^; and 56 mg L^−1^) for 7 and 10 days. The first and third sets of Figures **(A–I, S–AA)** show the cultivation in flasks, while the second and fourth sets **(J–R, AB–AJ)** show morphological alteration of the boron toxicity in the fronds. Bars represent a length of 5 cm (flasks) or 1 cm (plants).

**Figure 2:**
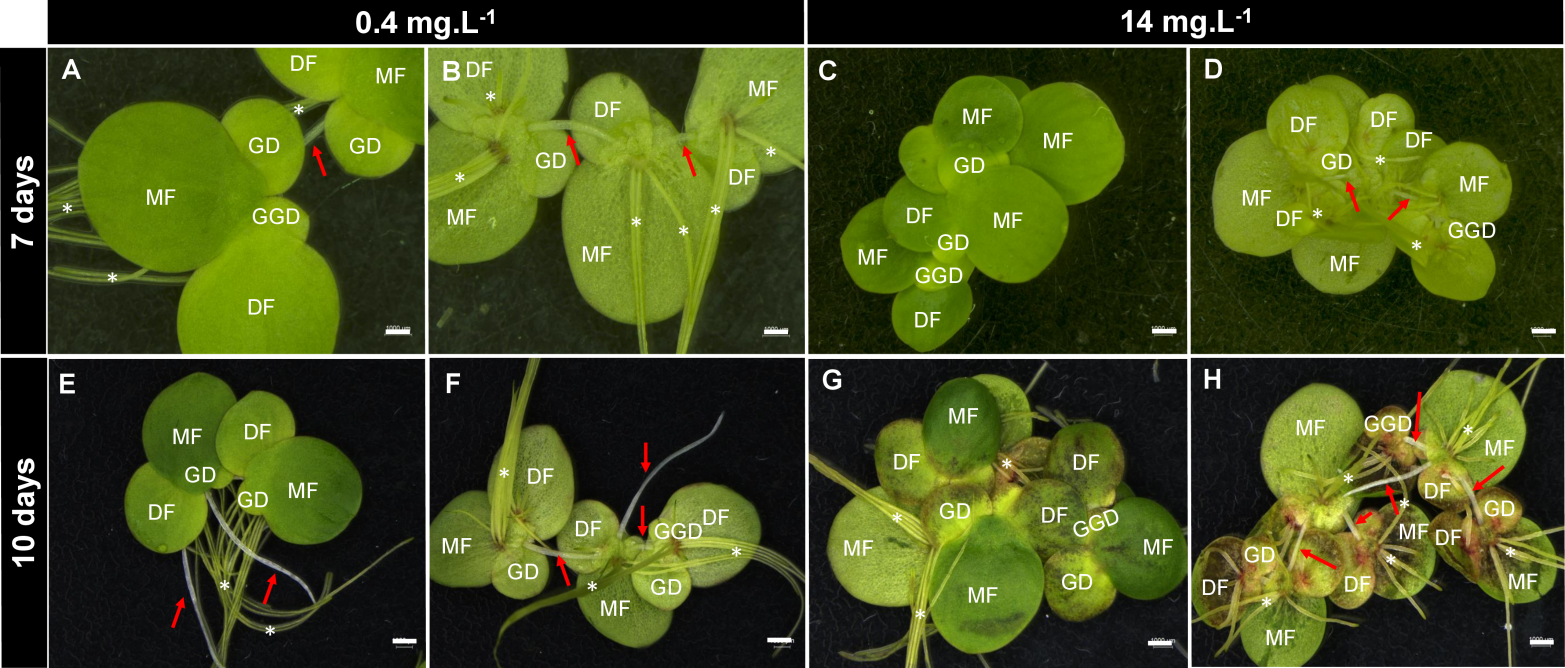
Effects of boron toxicity in *Spirodela polyrhiza*. Figures **(A, C, E, G)** show the abaxial view of fronds. Figures **(B, D, F, H)** show the adaxial view of fronds. *S. polyrhiza* clonal growth involves the development of new fronds from two lateral pockets, in which the new fronds are connected by the stipes (red arrows) that will elongate and release the new plant after full development. *S. polyrhiza* may have several roots (asterisks). MF, mother fronds; DF, daughter fronds; GD, granddaughter fronds; GGD, great-granddaughter fronds. Bars = 0.1 cm.

**Figure 3:**
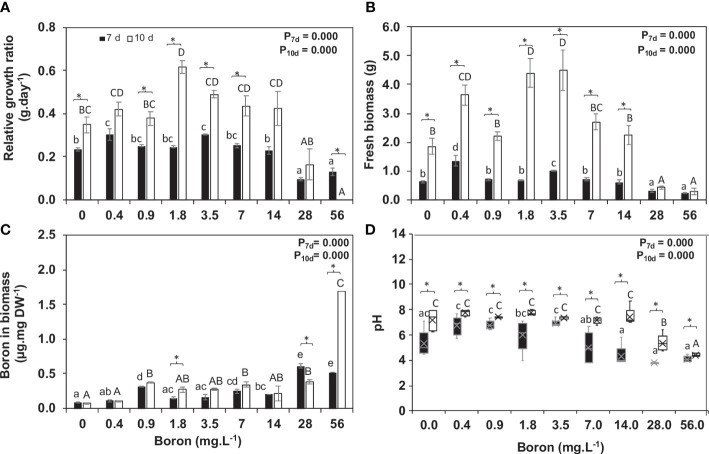
Boron uptake by *Spirodela polyrhiza* and its effect on relative growth ratio (RGR). **(A)** Relative growth rate (RGR), **(B)** fresh biomass accumulation at 7 and 10 days of cultivation, **(C)** boron content remaining in the media after 7 and 10 days of plant growth, and **(D)** pH of media after plant growth. Values shown are mean ± standard error (*n* = 5 for RGR, biomass, and pH, and *n* = 3 for boron in biomass). Black and white bars represent 7 and 10 days of cultivation, respectively. Significant differences among boron concentrations, using one-way ANOVA with Tukey´s test (*p*< 0.05), are shown by lowercase (7 days) and capital letters (10 days). Significant differences between 7 and 10 days of cultivation (for each concentration), using Student’s *t*-test (*p*< 0.05), are indicated by asterisks.

B concentrations altered the development of *S. polyrhiza*. Higher B levels (between 28 and 56 mg B L^−1^) led to chlorosis, dark leaf pigmentation, and reduction in growth (**Figures 1**, **2**, **3A**), suggesting toxicity. Plants grown at 0.4 mg B L^−1^ (control) had the highest growth rate and biomass accumulation at 7 days (**Figure 3A**). At 10 days, the highest growth and biomass accumulation was achieved at 1.8 mg B L^−1^ and maintained at 3.5, 7, and 14 mg B L^−1^ (**Figures 3A, B**). The highest *S. polyrhiza* growth was detected in 10 mg B L^−1^ after 10 days, in agreement with the higher B assimilation (**Figures 3A–C**). Besides that, in concentrations above 14 mg B L^−1^, the “daughter fronds” remained attached to the “mother frond” body causing colony over-integration (**Figures 1P, AH**, **2**). Therefore, under toxic conditions (above 28 mg B L^−1^), no stipe abscission (connectors from mother frond and daughter fronds) occurred (**Figures 2E–H**), which could not be recovered when these plants returned to the B concentration of the control (**Supplementary Figure 1**).

Plant boric acid assimilation is pH-dependent, altered during plant growth at 7 and 10 days. At 7 days, pH increased by 0.3 points from 0.4 to 3.5 mg B L^−1^ and got acidic (pH 4.2) from 7 to 56 mg B L^−1^, while at 10 days, the pH was somewhere neutral from 0 to 7 mg B L^−1^, getting acidic (pH 5.4 and 4.4) at the toxic concentrations (28 and 56 mg B L^−1^) (**Figure 3D**). B uptake from the media was on average 78%, and B content in *S. polyrhiza* biomass had minor alteration between 7 (0.08–0.52 µg mg^−1^) and 10 days (0.08–1.69 µg mg^−1^) with a tendency to increase over time (**Figure 3C**). *S. polyrhiza* under a toxic (28 and 56 mg B L^−1^) state accumulates 4.6 and 15 times more B than the control (0.4 mg B L^−1^) at 7 and 10 days, respectively (**Figure 3C**).

### 3.2 Non-structural carbohydrates signaling to the boron concentration and starch storage

**Figure 4:**
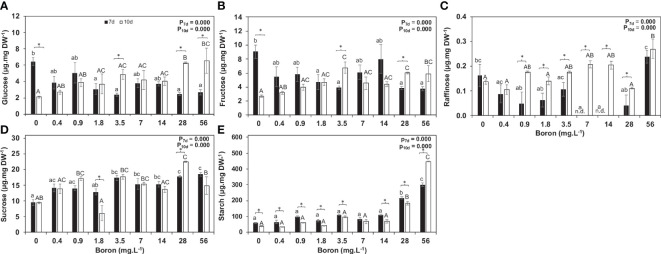
Non-structural carbohydrates and expression of *Sucrose synthase* and *Starch synthase* in *Spirodela polyrhiza* grown in different boron concentrations. Figures **(A-E)** show glucose, fructose, raffinose, sucrose, and starch levels, respectively, in µg mg^−1^ of dry weight (DW). Values shown are mean ± standard error (*n* = 5). Black and white bars represent 7 and 10 days of cultivation, respectively. Significant differences among boron concentrations, using one-way ANOVA with Tukey´s test (*p*< 0.05), are shown by lowercase (7 days) and capital letters (10 days). Significant differences between 7 and 10 days (for each concentration), using Student’s *t*-test (*p*< 0.05), are indicated by asterisks.

The non-structural carbohydrates evaluated are the first level of response to environmental changes during plant development and growth. Glucose content increased over time at B concentrations of 3.5, 28, and 56 mg B L^−1^ (**Figure 4A**), and fructose was reduced by 17% at high B at 10 days (**Figure 4B**). Sucrose levels were overall similar at 7 and 10 days, except for a twofold decrease in 10 mg B L^−1^ and a 25% increase in 160 mg B L^−1^ (**Figure 4D**) at 10 days. The sucrose increase might result from a slower consumption for growth as biomass accumulation and RGR were significantly reduced in this B concentration (**Figures 3A, B**). Raffinose, a sugar related to stress, increased at 10 days when compared to 7 days in most B concentrations tested and peaked by threefold at 56 mg B L^−1^ (**Figure 4C**). Moreover, higher B availability resulted in starch accumulation (**Figure 4E**).

### 3.3 B modulates cell wall monosaccharides

**Figure 5:**
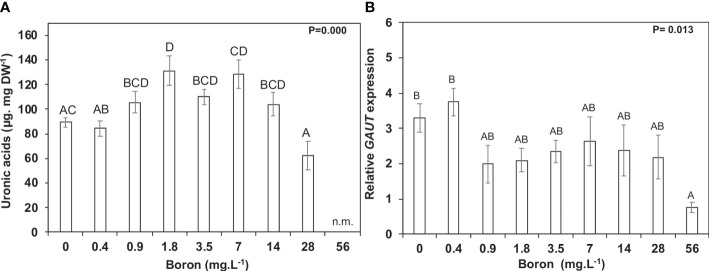
Pectin metabolism in *Spirodela polyrhiza* under boron toxicity. **(A)** Uronic acid contents at 10 days of cultivation. **(B)** Relative expression of *GAUT*, a gene responsible for synthesizing galacturonic acid chains in pectins. Values shown are mean ± standard error. Significant differences among boron concentrations, using one-way ANOVA with Tukey´s test (*p*< 0.05), are shown by lowercase (7 days) and capital letters (10 days). n.m. = not measured (*n* = 5).

Carbon allocation towards cell wall monosaccharides was altered in response to B availability during plant growth and development (**Tables 1**, **2**), mainly pectin modifications. This was unsurprising as *S. polyrhiza* has a rich pectic-cell wall, and B is strictly related to pectins. The concentrations from 0.9 to 14 mg B L^−1^ had an increase in uronic acids by 23.4% (104.1 µg mg^−1^ DW) to 55.4% (131.1 µg mg^−1^ DW), while 28 mg B L^−1^ B reduced pectic content (uronic acids) by 26.3% (62.2 µg mg^−1^ DW) when compared to 0.4 mg B L^−1^ (84.3 µg mg^−1^ DW) (**Figure 5A**). One of the genes related to the pectin scaffold to form homogalacturonan is α-1,4-D-galacturonosyltransferase (*GAUT - Spipo12G0021200)*, whose relative expression was higher at 0.4 mg B L^−1^ (**Figure 5B**).

**Table 1:** Cell wall monosaccharides of Spirodela polyrhiza grown in different boron concentrations for 7 days.

Boron (mg L^−1^)	Cell wall monosaccharides (µg mg^−1^)—7 days							
	Fucose	Arabinose	Galactose	Rhamnose	Glucose	Xylose	Mannose	Apiose
**0**	15.3^b^	102.6^ab^	164.0 ^ab^	39.5 ^b^	47.5 ^ab^	75.2 ^b^	99.4 ^b^	167.6^c^
**0.4**	7.4 ^ab^	76.3^b^	133.4 ^ab^	29.5 ^ab^	45.4 ^ab^	58.2 ^ab^	84.1 ^ab^	130.0 ^bc^
**0.9**	6.4 ^a^	98.3^ab^	150.1 ^ab^	30.7 ^ab^	64.8 ^ab^	53.9 ^ab^	83.0 ^ab^	88.4 ^ab^
**1.8**	6.1 ^a^	83.4 ^a^	128.2 ^a^	28.0 ^ab^	41.5 ^a^	54.7 ^ab^	73.2 ^a^	111.1 ^ab^
**3.5**	4.6^a^	82.2 ^a^	132.1 ^ab^	28.3 ^ab^	49.4 ^ab^	53.2 ^ab^	84.6 ^ab^	110.1 ^ab^
**7**	2.7^a^	83.7^a^	127.2 ^a^	23.8 ^a^	43.1 ^a^	43.9 ^a^	82.5 ^ab^	88.4 ^ab^
**14**	3.3^a^	91.5^a^	129.5 ^a^	23.7 ^ab^	50.8 ^a^	45.7 ^ab^	85.7 ^a^	74.6 ^ab^
**28**	3.8^a^	91.4^a^	138.1^ab^	21.6 ^a^	68.5 ^b^	41.1 ^a^	95.5 ^ab^	64.0 ^a^
**56**	5.9^a^	129.7^b^	170.0 ^a^	27.3 ^ab^	137.4 ^c^	52.9 ^ab^	84.6 ^ab^	89.0 ^ab^
***p-*value**	**0.005**	**0.000**	**0.006**	**0.013**	**0.000**	**0.007**	**0.006**	**0.000**

Values shown are mean ± standard error (*n* = 5). Significant differences among boron concentrations, using one-way ANOVA with Tukey´s test (*p*< 0.05). The darker the green, the higher the sugar levels.

**Table 2:** Cell wall neutral monosaccharides of *Spirodela polyrhiza* grown in different boron concentrations for 10 days.

Boron (mg L^−1^)	Cell wall monosaccharides (µg mg^−1^)—10 days							
	Fucose	Arabinose	Galactose	Rhamnose	Glucose	Xylose	Mannose	Apiose
**0**	6.8^a^	85.8 ^a^	137.2 ^ac^	29.6 ^a^	28.9 ^ab^	56.8 ^a^	84.1 ^ab^	151.4 ^b^
**0.4**	6.0 ^a^	74.6 ^a^	122.8 ^a^	27.0 ^a^	23.7 ^a^	54.0 ^a^	70.6 ^a^	137.2^b^
**0.9**	3.7 ^a^	116.6 ^ab^	152.9 ^ac^	32.2 ^a^	48.1 ^ab^	54.0 ^a^	72.4 ^a^	110.1^ab^
**1.8**	4.2 ^a^	86.0 ^a^	133.5 ^ab^	30.4 ^a^	34.3 ^ab^	57.3 ^a^	70.3 ^a^	147.3 ^b^
**3.5**	4.2 ^a^	78.3 ^a^	138.3 ^ac^	29.8 ^a^	51.4 ^ab^	57.7 ^a^	74.9 ^a^	138.0 ^b^
**7**	5.1 ^a^	103.9 ^ab^	145.6 ^ac^	32.7 ^a^	41.5 ^ab^	57.7 ^a^	66.6 ^a^	114.8 ^a^
**14**	7.8^ab^	150.1 ^bc^	189.3 ^c^	40.6 ^ab^	68.5 ^b^	62.4 ^a^	78.0 ^a^	109.2^ab^
**28**	12.4 ^b^	200.2 ^c^	253.5 ^d^	51.4 ^b^	113.0 ^c^	64.6 ^a^	66.9 ^a^	113.6^ab^
**56**	3.1^a^	152.4^bc^	179.2^bc^	28.3^a^	173.8^d^	49.1^a^	81.8^a^	65.4^a^
***p*-value**	**0.000**	**0.000**	**0.000**	**0.000**	**0.000**	0.619	0.664	**0.000**

Values shown are mean ± standard error (*n* = 5). Significant differences among boron concentrations, using one-way ANOVA with Tukey´s test (*p*< 0.05). The darker the green, the higher the sugar levels.

Rhamnose and apiose are monosaccharides characteristic of pectins and essential for plant growth and development. In different B concentrations, rhamnose content did not change at 7 days (**Table 1**). However, rhamnose increased by 12% at 14 and 56 mg B L^−1^ at 10 days when compared to 0.4 mg B L^−1^ (**Table 2**). Apiose is a B-linking sugar found in high levels in *S. polyrhiza*, and its content was reduced by 30% (7 days) and 17% (10 days) under high B (>7 mg B L^−1^) (**Tables 1**, **2**). Interestingly, at B deprivation (0 mg B L^−1^), the apiose levels were the highest identified for both 7 (151.4 µg mg^−1^ DW) and 10 (167.6 µg mg^−1^ DW) days (**Tables 1**, **2**). Besides that, the concentrations 0.4–7 mg B L^−1^ have an elevated biomass accumulation and UDP-apiose/UDP-xylose synthase (*AXS - Spipo0G0011100*) expression (**Supplemental Figure 2**), suggesting that the apiose levels might be related to *S. polyrhiza* growth capacity. The monosaccharides arabinose, galactose, and glucose increase in elevated B concentrations (**Tables 1**, **2**).

## 4 Discussion

Duckweeds have been reported as excellent phytoremediators (Oron et al., 1987), with a great capacity for B accumulation in their tissues (Frick, 1985). Here, the toxicity for the ecotype *S. polyrhiza* 9509 was found in the B concentrations above 28 mg B L^−1^ with the consumption of 78% of the B available on the growth media and accumulation up to 1.69 µg mg^−1^ in cell tissues (**Figure 1**). Furthermore, morphological changes in colony size were observed in high B concentrations due to the non-disruption of the stipe linking “mother frond” to the “daughter and granddaughter fronds” (**Figure 2**). An inverse phenomenon is reported for heavy metals (copper, silver, cadmium, nickel, zinc, aluminum, mercury, and chromate), which promote abscission in an early development stage and lead to colony disintegration by the middle lamella disruption in duckweeds (Li and Xiong, 2004; Topp et al., 2011). The reminiscence of the stipe is described for the first time (**Figure 2**). We hypothesized that the non-release of the daughter and granddaughter fronds controls the number of new fronds generated, consequently altering growth parameters. Other visible alterations were pigment intensification, necrosis, chlorosis, and frond death (**Figure 1**), corroborating previous findings (Davis et al., 2002; Villavicencio et al., 2007; Gür et al., 2016).

Abiotic stress, such as B toxicity, causes alteration in soluble sugar and starch levels in plants (Rosa et al., 2009; Lemoine et al., 2013). The increase of B in the cells promotes osmotic imbalances and a surplus of oxygen-reactive species (Reid, 2007; García-Sánchez et al., 2020) that can be overcome by the non-structural carbohydrates (Parida et al., 2002; Herrera-Rodríguez et al., 2010; Khatar et al., 2017). Fructose levels were higher under both B deprivation (0 mg B L^−1^) and toxicity (28 mg B L^−1^). At the same time, glucose revealed an inverse pattern in 7 and 10 days, with accumulation in 10 days in higher B concentrations (**Figures 4A, B**), and more raffinose is synthesized (threefold increase) at 56 mg B L^−1^ (**Figure 4C**). These elevated raffinose levels possibly signal stress, coordinate membrane trafficking and mRNA transport, and act as antioxidants and osmoprotectants (Hannah et al., 2006; Nishizawa et al., 2008; Elsayed et al., 2014).

The total B of the plant biomass is 70–90% related to the pectin linkages (Kobayashi et al., 1996; O’Neill et al., 2001; Riaz et al., 2021), and these polysaccharides act as regulators of the gradient balance of the B uptake to supply the plant requirement (Brown and Hu, 1994; Hu et al., 1996). The primary walls of grassy monocots have low pectin and low B requirement (3–10 B µg g^−1^ DW), while the walls of eudicots have higher pectin content and a greater need for B (20–30 B µg g^−1^ DW). Pteridophytes, lycophytes, and bryophytes have 21, 15.1, and 12.4 µg B g^−1^ DW, respectively (Matsunaga et al., 2004). Despite its low availability in the water, aquatic plants have a higher B requirement (Lemarchand et al., 2000). At 10 days of growth, the uptake of boric acid by S*. polyrhiza* was 76%, which accumulated into the biomass (0.08–1.69 µg B mg^−1^) (**Figure 3C**) and will be used for growth. Other aquatic plants such as monkeyflower (*Mimulus guttanus*) and fuzzy water clover (*Marsilea drummondii*) can accumulate up to 1,000 mg B kg^−1^ DW (Qian et al., 1999), which is lower than the capacity of *Lemna minor* (800–1,600 B µg g^−1^ DW) (Frick, 1985).

Pectic polysaccharides are an alternative and co-extensive network that transmits environmental signals to cells and joins the cell walls by the middle lamella (Fleischer et al., 1998; Matsunaga et al., 2004). Thus, pectins have an essential role in maintaining the wall architecture and plant defense. The control of the pectin content of *S. polyrhiza* may be related to B uptake, which at a toxic point (>28 mg B L^−1^) resulted in growth reduction and a decrease in pectin content by 26%. In contrast, the opposite was observed at 0.4 to 7 mg B L^−1^ (**Figure 5A**). The RGR reduced above 28 mg B L^−1^ (**Figure 3A**), which seemed much higher than reported for other duckweeds (6–16 mg B L^−1^) (Davis et al., 2002; Gür et al., 2016). Furthermore, previous studies demonstrated that higher B concentrations alter the wall’s porosity and tension properties, leading to cell elongation and changes in the cellular form (Matoh and Kobayashi, 1998; Camacho-cristóbal et al., 2008). The glucose, galactose, and arabinose contents increased by 633%, 46%, and 104%, respectively, at 10 days in 56 mg B L^−1^ (**Table 2**), suggesting alterations in hemicelluloses and pectin branching. Apiose, the main sugar of *S. polyrhiza* cell wall and a B anchor site, was reduced to 68% and 47% at 7 and 10 days in 56 mg B L^−1^ compared to 0.4 mg B L^−1^ (**Tables 1**, **2**). The gene responsible for synthesizing UDP-apiose, the nucleotide-sugar donor of apiose found in the pectins rhamnogalacturonan II and apiogalacturonan, is *UDP-apiose/UDP-xylose synthase* (*AXS*), which is essential for plant growth and development (Ahn et al., 2006; Zhao et al., 2020). *SpAXS* had elevated relative expression at 7 days in *S. polyrhiza* grown under different B concentrations, peaking at 1.8 mg B L^−1^ (**Supplemental Figure 2B**). This peak of SpAXS expression matches the highest growth rate and biomass accumulation, B uptake, and pectin content (**Figures 3** and **5A**). However, this higher expression pattern is not correlated with the apiose level itself, suggesting a fine post-transcriptional control. A similar trade was identified for xylose, another pentose found in the xylogalacturonans (pectins), and hemicelluloses (**Tables 1**, **2**, and **Supplemental Figure 2C**) related to the glycomic code evolution of *Lemnaceae* (Buckeridge, 2017; Avci et al., 2018).

Previous work from our group described a correlation between apiose, growth, and starch accumulation in duckweeds (Pagliuso et al., 2018). At the toxic point (28 mg B L^−1^), *S. polyrhiza* reduced the apiose content by 50.8% and 17.2% at 7 and 10 days, respectively, when compared to the control (0.4 mg B L^−1^). This reduction is hypothesized as a mechanism to reduce the B content stored in the plant tissues and block B uptake. As apiose has a trade-off with starch accumulation and growth, the starch was investigated. The starch accumulation was prominent in 7 days, especially for 28 and 56 mg B L^−1^ (**Figure 5E**), suggesting a faster plant response to accumulate more starch at high B concentrations. Starch accumulation and cell wall remodeling are mechanisms known in duckweeds to adapt and survive climatic changes and water-freezing surfaces (Longland et al., 1989). These modifications led to the formation of turions, a starch-rich frond with no aerenchyma presence and a distinct cell wall (Longland et al., 1989). Therefore, the increased starch and glucose reduced apiose and xylose as a response to the higher B uptake and colony alteration, and frond reduction might be mimetizing the turion formation to escape B toxicity.

## 5 Conclusion

Carbon allocation (non-structural and structural carbohydrates) changed under B deprivation and toxicity in *S. polyrhiza*. The toxicity for ecotype 9509 was determined at 28 mg B L^−1^, which led to non-disruption of the stipes linking the plant fronds, which, in turn, caused colony over-integration and changes in growth and development. The cell wall monosaccharides arabinose, glucose, and galactose had their content increase in elevated boron with a concomitant reduction of apiose, xylose, and uronic acids, suggesting modification on the ramifications of pectins and main hemicelluloses of *S. polyrhiza* along with the increase of starch. The carbohydrate modifications decreased the assimilation of boron probably overcoming toxicity.

## Data availability statement

The raw data supporting the conclusions of this article will be made available by the authors, without undue reservation.

## Author contributions

DP, AG, and MB planned and designed the experiment. DP, AG, and JP performed the experiments. JU and MC performed the boron quantification. DP and AG analyzed the data. DP, AG, and MB wrote the original draft, reviewed it, and edited it. All authors contributed to the article and approved the submitted version.
