# Supplementary material for: Carbon allocation of Spirodela polyrhiza under boron toxicity
Source: Front Plant Sci. 2023 Jul 17;14:1208888. doi: 10.3389/fpls.2023.1208888 (PMC10388368; doi:10.3389/fpls.2023.1208888)
Supplement: Supplementary file 1 [file DataSheet_1.docx]

Supplementary Material

Carbon allocation of *Spirodela polyrhiza* under boron toxicity

**Débora Pagliuso^1^, João Pedro de Jesus Pereira^1^, João Cristiano Ulrich^2^, Marycel Elena Barbosa Cotrim^2^, Marcos S. Buckeridge^1^, Adriana Grandis^1*^**

*** Correspondence:** agrandis@usp.br


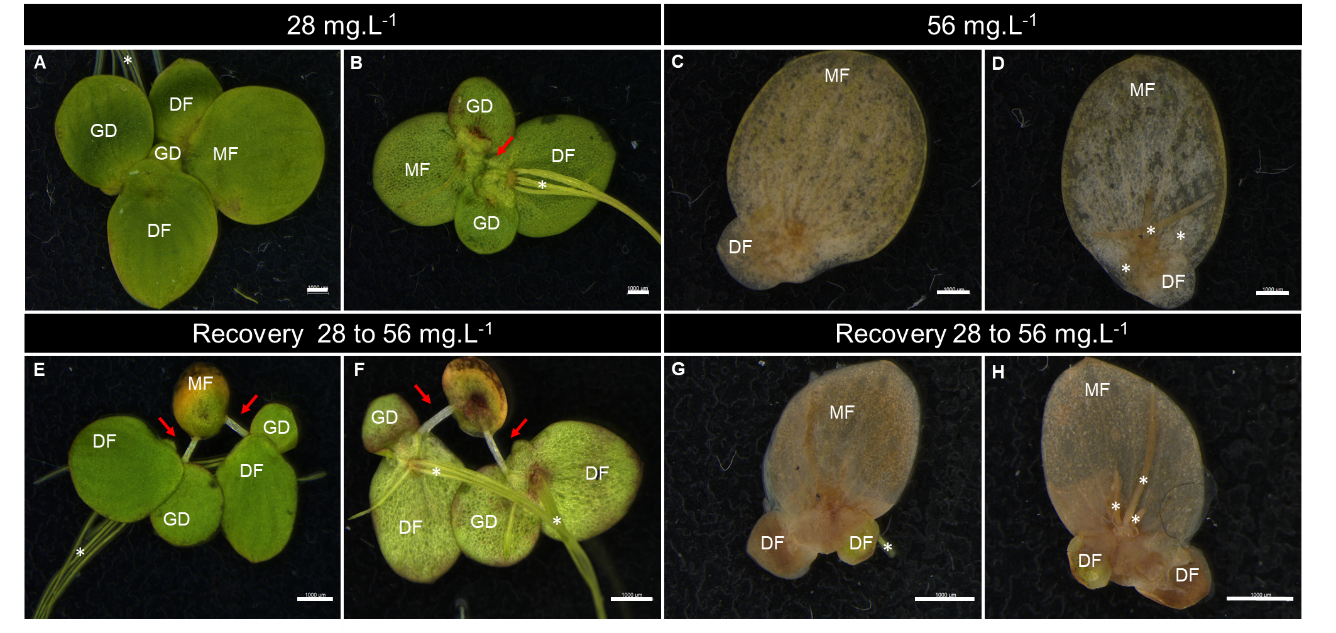


**Supplementary Figure 1. Boron toxicity recovery in *Spirodela polyrhiza*.** Plants were grown for 10 days at 28 or 56 mg.L^-1^ B before transference to media containing 0.4 mg.L^-1^ B. Plants were evaluated 30 days after the transference for recovery from damages caused by high B levels. Figures A, C, E, and G show the adaxial view of fronds. Figures B, D, F, and H show the abaxial view of fronds. Red arrows and asterisks indicate the stipes and roots, respectively. MF = mother frond, DF = daughter frond, GD = granddaughter frond. Bars = 0.1 cm.

**Supplementary Figure 2. Expression of genes related to pectin metabolism.** (A) Relative expression of *rhamnose biosynthesis* (*RHM*), a gene involved in rhamnose synthesis. (B) Relative expression of *UDP-apiose/UDP-xylose synthase* (*AXS)*, a gene involved in the apiose synthesis. (C) Relative expression of *UDP-glucuronate decarboxylase* (*UXS)*, a gene involved in xylose synthesis. Values shown are mean ± standard error (n = 5). Black and white bars represent 7 and 10 days of cultivation, respectively. Significant differences among boron concentrations, using ANOVA one-way with Tukey´s test (p<0.05), are shown by lowercase (7 days) and capital letters (10 days). Significant differences between 7 and 10 days (for each concentration), using Student’s t-test (p<0.05), are indicated by asterisks.

**Supplementary Table 1. Primer sequences used in this study.**

| **Gene** | **Transcript** | **5´-Foward primer-3´** | **5´-Reverse primer-3´** |
| --- | --- | --- | --- |
| Elongation factor 1-α (*EF1*) | Spipo2G0041800 | GGACCTGAAGAGAGGGTTCG | ACTTGACGGCGATGTGGG |
| F-BOX Family protein (*FBOX*) | Spipo5G0021900 | CCCACCAAGAACAGTCGCTT | TCAGATAATGCCCGCCGGTT |
| UDP-glucose-4,6-dehydratase (*RHM*) | Spipo0G0113900 | ACCCGGTCCTTCCATAAATC | AGTTCGATGGAGCAGAGGAA |
| UDP-glucuronate decarboxylase (*UXS*) | Spipo23G0021600 | CTCTGCCGGAAGTCCTCTT | GTTGAACGTGGTGGAGAACA |
| UDP-apiose/UDP-xylose synthase (*AXS*) | Spipo0G0011100 | ATACAGCCCTCAAACCGCAA | TTCTACGGGGAAGGCTACGA |
| α-1,4-galacturonoyil transferase (*GAUT*) | Spipo12G0021200 | TTCTACCTCCCCGAGGTCTT | TCGTCCAAGAAGAGCACCTT |
